# The Role of Flexibility in the Bioactivity of Short α-Helical Antimicrobial Peptides

**DOI:** 10.3390/antibiotics14050422

**Published:** 2025-04-22

**Authors:** Daniel Balleza

**Affiliations:** Laboratorio de Microbiología, Unidad de Investigación y Desarrollo en Alimentos, Instituto Tecnológico de Veracruz, Tecnológico Nacional de México, Veracruz 91897, Mexico; daniel.bm@veracruz.tecnm.mx

**Keywords:** short antimicrobial peptides, intrinsic flexibility, dipolar moment

## Abstract

The formation of aqueous pores through the interaction of amphipathic peptides is a process facilitated by the conformational dynamics typical of these biomolecules. Prior to their insertion with the membrane, these peptides go through several conformational states until they finally reach a stable α-helical structure. The conformational dynamics of these pore-forming peptides, α-PFP, is, thus, encoded in their amino acid sequence, which also predetermines their intrinsic flexibility. However, although the role of flexibility is widely recognized as fundamental in their bioactivity, it is still unclear whether this parameter is indeed decisive, as there are reports favoring the view of highly disruptive flexible peptides and others where relative rigidity also predetermines high rates of permeability across membranes. In this review we discuss in depth all those aspects linked to the conformational dynamics of these small biomolecules and which depend on the composition, sequence and dynamic performance both in aqueous phase and in close interaction with phospholipids. In addition, evidence is provided for the contribution of the known carboxyamidation in some well-studied α-PFPs, which are preferentially associated with sequences intrinsically more rigid than those not amidated and generally more flexible than the former. Taken together, this information is of great relevance for the optimization of new antibiotic peptides.

## 1. Introduction

Protein flexibility is a topic of intense debate. Typically, this structural parameter is associated with a high conformational freedom of the polypeptide chain [1], the presence of intrinsically disordered regions [2] or a high rate of atomic thermal fluctuation [3,4]. However, in short sequences of amino acid residues, i.e., oligopeptides, the effect of flexibility must be enhanced since complete transitions from disordered states to ordered configurations (alpha or beta) frequently take place in strong relation with the size of the chain [5]. Furthermore, it is now established that this all-or-none structural reconfiguration is strongly influenced by solvent conditions [6,7,8] and the intrinsic propensity to stabilize secondary structures in terms of the chemical composition [9]. Thus, considering these factors in the context of the mechanisms of action of antimicrobial peptides (AMPs) is crucial.

AMPs are widely distributed along the tree of life, being present as part of the defense system of animals, fungi and plants. Given this biological diversity, it has been proposed that multicellular organisms express antimicrobial peptides as a key element of their immune system [10,11]. As a general rule, these peptides consist of a few amino acids (even fewer than 10 residues) and up to about 50 residues with net positive charge at physiological pH. Their chemical composition is amphipathic, and they do not have a defined composition since they are usually very diverse in nature. Some peptides are linear α-helical, while others preferentially form β-sheets and include disulfide bridges, but cyclic peptides and peptides with extended flexible loop structures have also been described [11]. AMPs are classified based on (i) source, (ii) activity, (iii) structural features and (iv) amino acid-rich composition [12]. However, it is important to distinguish also their mechanisms of action, since some of them may include activities that interfere with intracellular functions, by inhibiting DNA, RNA or protein synthesis, but a large number of AMPs have evolved as bioactive agents targeting cell membranes [13]. Herein, I have focused this study on peptides capable of forming pores in membranes, specifically, the so-called α-helical ‘pore-forming peptides’ (α-PFP), as a distinction from those known as pore-forming-sheet peptides [14] or cell-penetrating peptides [15].

Since their discovery and isolation from diverse biological sources, intensive research efforts have been made to understand the molecular mechanisms that facilitate the antimicrobial actions of these biomolecules. One of the best studied peptides and also the most reported in the literature to date (almost 2900 articles and reviews in PubMed) is melittin (MLT), a cationic peptide consisting of 26 amino acid residues (GIGAVLKVLTTGLPALISWIKRKRQQ-NH_2_). Since its discovery, it became clear that the pharmacological potential of MLT was huge, given that by inserting into lipid membranes and co-associating with other peptides of the same nature, MLT is able to form transient aqueous pores and facilitating membrane permeabilization [16,17,18,19]. Under these circumstances, MLT and other α-PFP are able to knock down the osmotic balance of target cells and cause the death of several bacterial pathogens [20,21].

With all this information, it soon became necessary to establish the laws and principles governing the bioactivities of this class of peptides from a molecular perspective. To this end, MLT became a paradigm of biomembrane biophysics for the study of the physicochemical properties of α-PFP, as well as the study of the contribution of lipid composition in their bioactivities, and the structural aspects related to the interaction between those peptides and the lipid bilayer. From this perspective, one of the aspects that has generated more questions has been the nature of the aqueous pores that α-PFP are capable of generating. MLT forms stable water pores of 4.4 nm in diameter as determined by neutron in-plane scattering and an architecture consistent with the toroidal model [22]. However, there is also evidence of the probable additional effects of some collateral effects as MLT internalize and carry out their effects more independently of the lipid membrane, for example, inducing depolarization of the mitochondrial membrane, which affects ATP production and destroys mitochondria [23]. In this review, the events that will be discussed in depth are those directly associated with the interaction, adsorption and translocation of α-PFP in phospholipid bilayers, events that facilitate the formation of aqueous pores or promote the disintegration of the membrane by micellization processes.

The formation of aqueous pores in lipid bilayers is a fairly common event. Given the non-covalent nature of the interactions in the lipid matrix, phospholipids are subject to thermal fluctuations, packing defects and the formation of transient water wires, also called hydration defects [24,25]. However, the frequency of these defects increases significantly in the presence of the adsorbed, translocated and incorporated peptides along the lipid bilayer normal [26]. Thus, α-PFPs form dynamic supramolecular assemblies in association with lipids and are able to stabilize transmembrane pores or even to completely disrupt the lipidic membrane in a dose-dependent manner [27,28].

From a more mechanistic point of view, aqueous pore stabilization as a function of α-PFP flexibility has recently been established, although many molecular details remain to be resolved. In this context, an important contribution of the flexibility and propensity of certain peptides to acquire kinked structures has recently been reported. This facilitates the potential deformability of the bilayer and the stabilization of pores with toroidal geometry [28]. In the following sections, we will develop these topics in more detail, emphasizing those aspects that are still poorly understood.

## 2. Peptide Order and Dipolar Moment (DpM)

**Figure 1:**
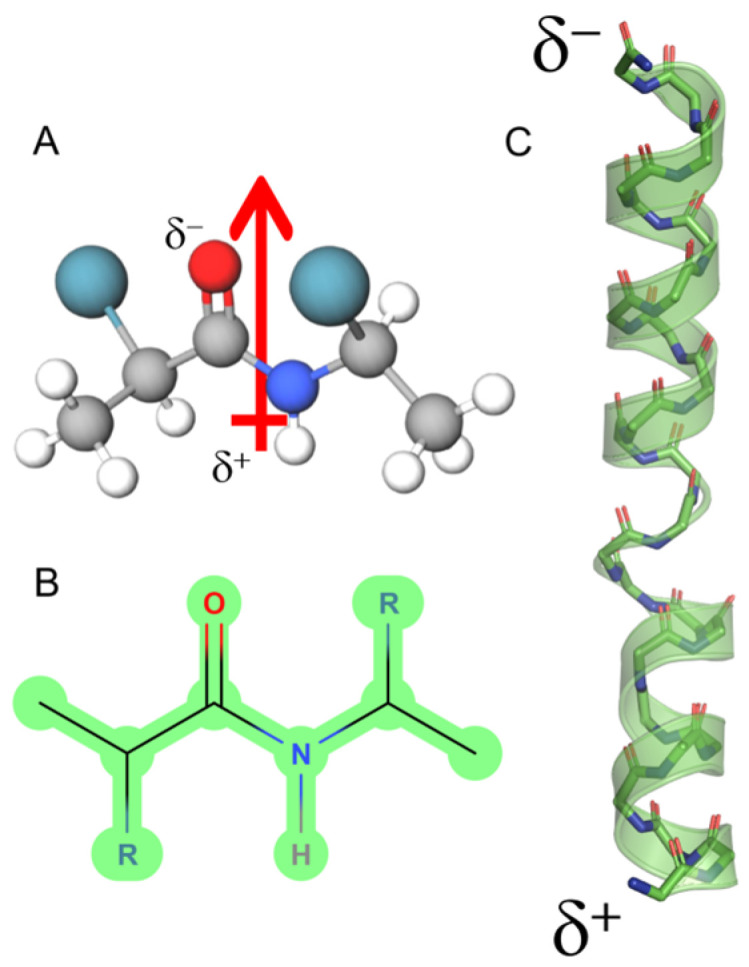
Dipole moment of an α-helix peptide. (**A**) A peptide unit showing the orientation of the dipole vector (arrow). (**B**) The peptide bond in Fisher projection. (**C**) Schematic view of the macrodipole. The total assembly of the α-helix produces a macrodipole where the N-end exhibits a positive partial charge (δ^+^), whereas the C-end exposes a negative partial charge (δ^−^). The solved structure for melittin is depicted (PDB ID:2MLT).

The typical conformation adopted by short amino acid sequences of α-PFPs is the 3.6_13_ right-handed helix. This helical configuration maximizes the intramolecular hydrogen bonding. Unlike the beta (β) strand configuration, where hydrogen bond formation alternates between the carbonyl and amino groups of the main chain, in α-helices, the formations of H-bonds all point in the same direction [29]. This structural peculiarity significantly contributes to the creation of a dipolar vector along the folded helix [30] (Figure 1). In comparison, although there is evidence that certain α-PFPs such as MLT and UyCT1 (GFWGKLWEGVKNAI-NH_2_) can be configured to form beta structures [31,32], the helical content estimated for this peptide by circular dichroism is >60% in model liposomes that simulate the lipid composition of bacterial (POPE:POPG, 70:30) or mammalian cells (POPC:Chol, 8:2) [33]. Thus, since the alignment of each dipolar vector along the α-helix is unidirectional, the contribution of this physico-chemical parameter to the degree of order of that structure is decisive. In that sense, the conformational freedom of the helical structure is less prominent compared to the beta configuration, which, in principle, would make the helices present in these short peptides significantly more ordered structures with fewer intramolecular contacts. 

**Figure 2:**
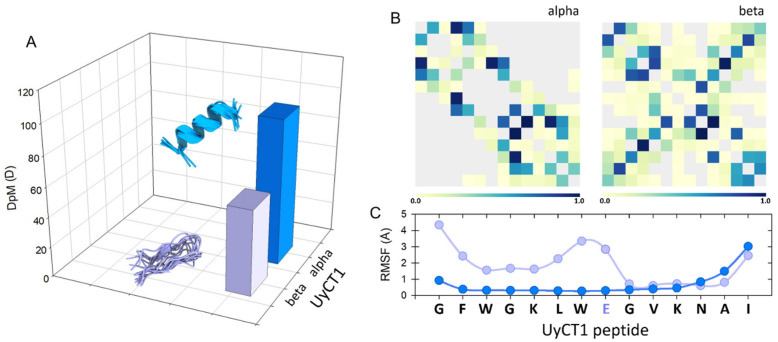
(**A**) Conceptual representation of the magnitude of the dipole moment (DpM) of the UyCT1 peptide as a typical α-helix (blue) or a β-sheet (light violet), where the DpM is significantly reduced from μ = 96.8 ± 20 D to μ = 56.8 ± 10.3 D. (**B**) Intramolecular contacts in alpha (left) and beta (right) configurations. (**C**) Atomic fluctuations (RMSF) mainly in side chains; in this peptide, the presence of a glutamate residue near the center of the structure, a residue of high intrinsic flexibility, favors a higher degree of atomic fluctuations towards the N-end of this peptide.

Figure 2 depicts this; the UyCT1 peptide was subjected to the GROMOS96 43a1 force field and, as a result, the structure has the potential to generate multiple α-helical conformers, with a high DpM and a restricted number of contacts (panel B, left) compared to a lower propensity to acquire β-sheet configurations, with lower DpM and a higher number of intramolecular contacts (panel B, right). This behavior is typical for structures with high flexibility, which is determined by the root mean square fluctuation (RMSF) value, a measure of the average deviation of atomic positions from their mean positions over time in MD simulations. Thus, this structural parameter provides information about the flexibility and dynamics of a polypeptide structure, indicating that regions with higher RMSF values tend to be more flexible, while regions with lower values are typically more rigid. In the specific case of the UyCT1 peptide in beta configuration, the N-terminal half exhibits a greater propensity to disorder compared to the same peptide when forming α-helices (Figure 2C).

In that sense, α-PFP functionality depends in part on the contribution of the DpM, which has important effects on adsorption at the bilayer/water interphase, packing to form multimeric complexes, and the stabilization of multimers as transient aqueous pores through electrostatic and hydrophobic interactions. Taking, again, the case of MLT as a model, four phases involving the contribution of DpM during the interaction of this peptide with the membrane surface have been postulated [34]: (1) binding energy associated with electrostatic attraction between the negatively charged surface of the membrane and the six positive charges present in the MLT structure; (2) adsorption of one peptide molecule per 1270 Å^2^; (3) decay of the surface electrostatic potential of about 1/3 with respect to the free solution; and (4) rearrangements of phospholipids and surface water molecules as an effect of the DpM magnitude per bound peptide, which is achieved by tangentially orienting each dipole.

In a concentration-dependent effect of peptides attached to the surface, the reorientation of peptides is facilitated by translocating and penetrating the bilayer, which reduces their free energy [35,36]. In a synthetic peptide, MelP5 (GIGAVLLKVLLLTTGLPALISWIKAAQQQQ-NH_2_), 10-fold more potent than Mel, the formation of parallel-oriented hexameric pores has been shown to generate the toroidal geometry typical for this class of α-PFPs. In this orientation, the associated DpMs contribute to the stabilization of pores fluctuating around a radius of 14 Å, whereas the antiparallel orientation creates more compact structures without the significant participation of polar lipid heads, forming smaller aqueous pores with a radius of ~6 Å [37]. However, this may not be a general rule [36].

In any case, one of the main determinants to describe the activity of α-PFPs is their dipole moment, which is an indicator of the degree of order that the structure acquires once it is adsorbed and becomes ordered and translocation through the lipid membrane is facilitated. The binding of unstructured peptides is calculated using the Wimley–White interfacial scale [38]. However, in solution, it is very likely that the α-PFPs are in different conformations, including disordered loops, varying degrees of α-helicity and, to a lesser extent, some β-sheets. As the Gibbs free energy of membrane binding by these partially ordered peptide complexes in the aqueous phase is lower than that in the disordered conformations [39,40], their adsorption and subsequent insertion through the bilayer is favored; this reduces the Δ*G* of the interaction, permeabilizing the membrane [28].

## 3. Degree of Helicity and Penetrability in Lipid Bilayers

The most relevant event in terms of the bioactivity of α-PFPs is their insertion into the membrane. Once the first electrostatic recognition occurs, experimental evidence convincingly indicates that the structure of the α-PFPs changes significantly [31,41,42]. In the case of MLT and other AMPs, a multitude of studies indicate that the degree of helicity is directly associated with binding to model lipid systems and the general consensus is that a higher degree of intrinsic helicity (i.e., in the aqueous phase) typically implies a higher bioactivity in terms of the minimum concentrations needed to inhibit the growth of multiple bacterial pathogens [43,44,45]. Given the cationic nature of these peptides and in view of the fact that bacterial membranes are rich in phosphatidylglycerol (PG), one of the main challenges in terms of the use of various AMPs as potential antibiotics is how to discriminate PG-rich membranes of bacterial origin from those rich in the majority anionic lipid present in mammalian cells, i.e., phosphatidylserine (PS), excluding, in turn, zwitterionic lipids typical of these eukaryotic membranes, i.e., phosphatidylcholine (PC), phosphatidylethanolamine (PE) and sphingomyelin (SM). Recent studies have convincingly demonstrated the great effect that helicity has on this kind of molecular recognition. A new class of radially amphiphilic α-helical AMPs selectively discriminate PG-rich membranes from those enriched in PS. The group led by this author has been able to modulate, in a controlled manner, the degree of helicity in this class of peptides by incorporating new chiral centers in the peptide chains with D-amino acids and regulating their degree of polymerization [46]. It was, thus, proven that by increasing the degree of helicity, these so-called radially amphiphilic peptides (RAPs) interact better with POPG-rich liposomes, in addition to significantly enhancing their antimicrobial activity [47].

However, although some modified peptides possess a more ordered structure in terms of helical content in the aqueous phase, this does not necessarily imply a better bioactivity in the target cells. Hence, a preordered structure is not necessarily effective in killing bacteria. This has been observed, for example, through circular dichroism (CD) experiments, which indicate that MLT-P14C, a MLT variant, exhibits a high helical content both in the aqueous phase and in model bacterial membranes (POPE:POPG) and model mammalian membranes (POPC:Chol). Under such conditions, the MLT-P14C variant was able to more effectively permeabilize fluorescent dye-carrying liposomes with respect to the wild-type peptide (MLT) but the bioactivity of this modified peptide turned out to be significantly poor against several bacteria [48]. Clearly, at least for the case of MLT, the role of the central proline seems to be determinant.

**Figure 3:**
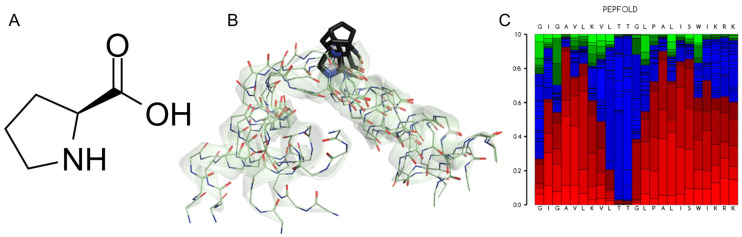
(**A**) L-proline, (2S)-pyrrolidine-2-carboxylic acid. (**B**) Five superimposed structures for Melittin generated by the PepFold server (https://bioserv.rpbs.univ-paris-diderot.fr, accessed on 12 December 2024) to reveal the Pro-kink motif. The role of the Pro-14 residue (black) stands out, which determines a high degree of potential mobility for the N-terminus of this peptide. (**C**) Graphical representation of the probabilities of potential substructures using the next code: helical (red), extended (green) and coil (blue).

Proline is an atypical amino acid. Actually, it is an imino acid, since it has a secondary amino group (imine) and not the typical primary amino group (Figure 3A). This peculiarity has important structural repercussions because the three-carbon R group is fused to the α-N, which results in the formation of a rigid ring with high rotational restriction. As a result, each prolyl residue imposes important restrictions in the folding of polypeptide chains [49]. This chemical geometry has provided much insight into the effects of the inclusion of this residue in the context of conformational freedom in many proteins. Therefore, proline has been considered both a flexibility-enhancing residue [50] and a flexibility-restricting one, depending on the molecular context [51]. Similarly, the segment bending motion observed in alamethicin can be attributed to the presence of the G-X-X-P motif in the central part of that peptaibol [49].

The role of Pro in the conformational dynamics that lead to peptides being either inserted into or stabilized by forming aqueous pores across the lipid bilayer has been studied in some detail. Again, what we know in this regard for MLT indicates that Pro14 facilitates peptide translocation and contributes significantly to the creation of aqueous ion-permeable pores by forming hinges that pack as helical bundles [52]. In any case, there is evidence of a high degree of mobility around the central proline of MLT [53], an observation that we have corroborated using modern computational tools such as PepFold (Figure 3B,C) [54]. Such experimental observations have also been corroborated by molecular dynamics (MD) simulations. These studies indicate that above a threshold, where a population of peptides is already adsorbed on the membrane surface, a cascade of insertion occurs through the defects inherent to the packing of phospholipids, leading to the formation of stable pores [55]; however, in this study it was not fully demonstrated that the central Pro residue at position 14 is an essential component of this proposed mechanism. Nevertheless, the role of a central Pro residue, perturbing the bilayer structure and facilitating the insertion of peptides such as Maculatin 1.1 (21-aa), Buforin II (21-aa) and Uy234 (18-aa), has been postulated elsewhere [56,57,58]. In any case, it is known that MLT, upon internalization into the bilayer due to its natural internal bending, acts as a wedge as it is adsorbed in the interfacial region, leading to a local thinning of the membrane and an asymmetry in the monolayers that has the potential to disintegrate them [59]. The structural impact of the bending effect of Pro14 facilitates the lytic activity of MLT since this residue affects the electrostatic properties of MLT and this prevents a shielding of their positively charged residues [60].

In these scenarios, α-PFPs would be able to take advantage of the packing defects present at the edges of the lipid domains, a situation that could favor the generation of the driving force necessary to open larger and larger pores [19], leading this kind of system to promote micellization phenomena or act as transient ionophores [61,62]. In other studies, it has been determined that the presence of this flexible kink (the so-called Pro-kink motif, Figure 3) in α-helical PFPs facilitates the formation of pores with toroidal geometry, destabilizing barrel-stave pores [63]. However, the presence of a central Pro residue may not be indispensable for this class of peptides, since it has also been shown that by replacing this residue, present in Pin2 (24-aa), with GV, VG or GVG hinges, a ‘flexibilizing’ effect analogous to that of the Pro-kink motif is obtained. This effect, in turn, corresponds quite well with the antibacterial activity in the resulting peptide variants [64]. In the case of Smp24 (24-aa), the result was, on the contrary, the opposite, as the substitution of the central Pro for a GVG motif downregulated its bioactivity against diverse pathogens, including both Gram-positive and Gram-negative bacteria [65]. Thus, the presence of flexible motifs analogous to the Pro-kink motif, such as GVG, must act differently mechanistically. In MD simulations, that variant (Pin2GVG) in POPC-based model lipid systems is not able to internalize properly and the interaction with the hydrophobic core is not favored [66]. The flexibility-enhancing effect induced by Gly has also been reported for variants of the synthetic peptide kiadin, where a high proportion of Gly has been shown to reduce their helicity, make them generally less potent and causing little disruption of integrity in bacterial and fungal membranes [67,68].

In Ranateurin-2CSa (GILSSFKGVAKGVAKDDLAGKLLETLKCKITGC), the presence of a tandem-turn-repeat from position G8 to K15 creates a helix-turn-helix interhelical deformation, whose maximum internal bending is formed at position K11-G12. This architecture facilitates the C-terminal segment of the peptide, characterized by the presence of a disulfide bridge, to maximize the amphipathic character of the entire segment and adopt kinked helical structures. It has been proposed that this intrinsic flexibilization facilitates the internalization of the peptide into anionic membranes, typical of bacteria, through an enhanced interaction with those interfaces [69,70,71].

Since their discovery, it has been speculated that the mode of action of α-PFPs includes a transition from an in-planar to a transmembrane configuration [72]. Reaching this conclusion has required years of intensive research, such that we now have convincing evidence of the molecular events that culminate in the formation and stabilization of aqueous pores across the lipid bilayer. By using solid-state NMR spectroscopy with ^15^N-labeled peptides and ^31^P-labeled lipids, it has been possible to obtain information on the spatial orientation of such helical structures and their impact on the ordering of the polar groups present in the lipids with respect to the membrane normal [73,74]. On the other hand, oriented circular dichroism (OCD) allows to quantify the energy associated with the electronic transition dipole moments of the amide bonds throughout the main chain. The interaction of the electric field vector of circularly polarized light with these electronic transitions makes it possible to determine the angle between the helix axis and the normal of the bilayer. With this innovative biophysical tool, it has been possible to establish the transition of the natural peptide PGLa (GMASKAGAIAGKIAKVALKAL-NH_2_) and the synthetic MAP peptide (KLALKLALKALKAALKLA-NH_2_) from a membrane-bound state, through various degrees of tilt during the insertion process, up to a transmembrane state completely immersed in the membrane [75]. The strongly cationic character of the PGLa peptide (net charge, NZC = +1/+4) is key to understanding the high bioactivity of this peptide against Gram-positive and Gram-negative bacteria [76].

A recent study has established that the importance of a flexible ‘tail’, in particular, the KAL-NH_2_ motif (K19, A20, L21), which actively participates in the spontaneous insertion of the peptide into the outer lipid monolayer, promotes its aggregation state and facilitates the formation of aqueous pores. The conformational adaptability of these residues enables this peptide to become increasingly inserted into the bilayer, with increases in its synchronous tilt, in turn coordinated with the associated bending of the carboxyamidated end [77]. It is tempting to speculate that the high flexibility of the KAL-NH_2_ motif is associated with the presence of a highly flexible residue such as K19 at a position where the stabilization of a last turn in the helix would possibly be compromised. This, in turn, would favor a close dependence on this residue as a rudimentary voltage sensor, regulating the insertion of this peptide as a function of the membrane potential. Indeed, there is clear evidence that the degree of tilt that the PGLa peptide assumes during its insertion into the membrane depends directly on the transmembrane potential and that high potentials promote an increase in the population of peptides with high degrees of tilting [78]. This is in agreement with experimental evidence indicating that hyperpolarizing potentials contribute to the degree of insertion of several cationic peptides [79].

On the other hand, the synergistic effects of cationic peptides such as PGLa or Mag2 have also revealed an interesting contribution of the hydrophobic moment vector to the permeability of these aggregates, since in equimolar combination, both peptides facilitate membrane permeabilization [80,81]. This is consistent with some relevant physicochemical parameters of both peptides, such as the hydrophobic moment (HM) [82]. The HM vector of Mag2 (9.65 kTÅ/*e*) is significantly higher than that of PGLa (5.5 kTÅ/*e*), which results in a stable perpendicular orientation with respect to the membrane normal for Mag2. On the contrary, the PGLa peptide achieves, in such conditions, a more transmembrane topology, even though this peptide has an extra positive charge with respect to Mag2 but exposes a larger hydrophobic face than that peptide, stabilizing it in the hydrophobic core of the membrane [83].

The use of computational tools has allowed to simulate the permeabilization mechanisms of several α-PFPs, forming nanoaggregates and inserting into the lipid bilayer to form transient aqueous pores. This type of study has achieved the corroboration of many experimental data, which has made it very useful as an additional tool in the rational design of peptides as potential antimicrobial drugs [19,77,84,85].

## 4. Pore Formation and Peptide Intrinsic Flexibility

Once inserted into the bilayer, several α-PFP peptides can follow one of three destinations: (1) stabilizing a toroidal pore, (2) structuring a barrel pore or (3) inducing a detergent-like effect on membranes. The formation of aqueous pores across membranes is a process directly associated with the free energy of the system, so that, considering a monomeric model for an α-PFP and the subdivision of the bilayer normal into two thin hydrophilic interfacial regions and a thicker hydrophobic core, two insertion paths can be considered. The first would be an aqueous pathway and the second an interfacial pathway. The free energy associated with folding and insertion along the aqueous pathway has two components, the change in free energy for the folding of a disordered peptide into an α-helical one (Δ*G_fw_*) and the change in free energy for the insertion of this ordered peptide into the apolar core of the membrane (Δ*G_whc_*) [86]. Once a sufficient number of peptides are inserted into the bilayer, e.g., four peptides for MLT [87], the energy barrier that must be overcome to open an aqueous pore is about 5 kT [88].

In that sense, as α-PFP are typically dynamic in solution, multiple conformational families coexist in interconvergent populations, including α-helical or β-sheet conformations, a mix of both or even a lack of secondary structures. These structural reconfigurations are associated with changes in energy landscapes linked to the nature of the aqueous solution and lipid composition [89]. This is crucial to understand the mechanisms of action where the membrane structure collapses in the presence of these peptides. Furthermore, the optimal length of a peptide to achieve opening is 4 nm (the typical membrane thickness). However, the energy minimum for peptides of 5–7 nm is essentially the same and once opened, the pore expands spontaneously as a function of the peptide-to-lipid (P/L) ratio, where the exergonic change of the process is accelerated as a function of the magnitude of this ratio [88,90].

In a strict dose/response effect, a minimum free-energy threshold must be reached to stabilize each type of aqueous pore (i.e., toroidal or barrel-stave type) prior to a very probable ‘detergent effect’ typical of the carpet mechanism reported by several authors [91,92,93]. This energetic threshold must be associated with the minimum number of α-PFPs indispensable to configure the aqueous access pathway from the pre-existence of the aqueous defects already mentioned above. Therefore, toroidal pores may be the most stable structure from an energetic point of view, in good agreement with the large amount of experimental evidence for some peptides where they have been described, including MLT [18,94], Magainin 2 (Mag2, GIGKFLHSAKKFGKAFVGEIMNS-NH_2_) [95,96], tritrpticin TRP3 (VRRFPWWWPFLRR-NH_2_) [97], Smp24 (IWSFLIKAATKLLPSLFGGGKKDS) [98], cathelicidin AMP LL-37 (LLGDFFRKSKEKIGKEFKRIVQRIKDFLRNLVPRTES) [99] and the bacteriocin lacticin-Q (53aa) [100]. On the other hand, the formation of barrel-stave pores may be energetically disadvantaged, since to date, it has only been described for peptaibol alamethicin (ALM) [101,102], antiamoebin I [103] and, probably, ceratotoxin A and protegrin-I [104,105].

**Figure 4:**
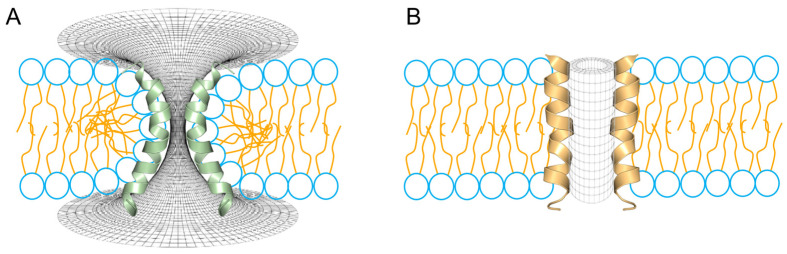
Two different topologies for the configuration of aqueous pores through lipid membranes. (**A**) Toroidal pore, a typical interpretation for the mechanism of action for melittin and magainins. (**B**) Barrel-stave pore, a typical configuration found in voltage-dependent ion channels. In the schematic representation of the phospholipids, the head groups are shown in blue and the peptides are shown fully helical for melittin (green) and alamethicin (orange).

The toroidal and barrel-stave transmembrane pore models differ in the orientation of the annular lipids in direct contact with the peptides. Given that lipids exhibit a high degree of atomic motility and deformability [106], the co-dependence of these nanostructures in terms of the flexibility of both counterparts is evident. In the toroidal model (Figure 4A), a higher degree of flexibility is required by the peptide component, flexibility that is in direct relation to the composition of the α-PFPs, and where residues of high intrinsic flexibility such as Gly, Pro and Glu, or polar residues such as Ser and Asn, are residues that are frequently found near the central helix region in a large number of short α-PFPs. We recently describe this for a set of more than 130 peptides present in the venoms of several scorpions, which integrate two major groups, i.e., the stigmurin-like and the pantinin-like peptides [107]. For this class of toroidal geometries, 6 to 12 monomers have been calculated, forming 20–40 Å anion-selective pores, with some peptides still attached to the pore edges in both interfacial regions [19]. Finally, although the free energy associated with the integrity of toroidal pores formed with MLT has been estimated around 22.4 kcalmol^−1^ [19], in good agreement with some studies where the temporal stability of MLT pores has been experimentally tested, eventually, these pores may collapse and disintegrate the membrane as a function of the P/L ratio [108].

The barrel-stave pore (Figure 4B) has been a more elusive structure and, unlike the toroidal model, it requires a lower degree of flexibility (i.e., is more rigid), which corresponds to the high degree of hydrophobicity, typical of ALM. Thus, in this class of peptide pores, the high proportion of hydrophobic residues allows the annular lipids to adjust the thickness of their hydrocarbon core to match the hydrophobic surface of the bundle of peptides [109]. Thus, the ALM pore has been widely accepted as a precellular model in the evolution of ion channels, since the structure consists of a cylindrical arrangement of parallel peptide helices with a central lumen filled with water [110]. Furthermore, the high proportion of hydrophobic residues in ALM facilitates peptide–peptide and peptide–lipid interactions, while excluding lipid head groups from the aqueous pore [111,112]. Furthermore, in terms of flexibility, helix bending, which is crucial in the case of the stabilization of toroidal pores, in barrel-stave pores it might not be so critical for the formation of such structures, in terms of their channel-like (voltage-dependent) activity, a typical behavior of ALM pores [113].

## 5. Micellization and the Detergent Effect

From an energetic perspective, the formation of ‘wormhole-like’ toroidal pores could be the most common fate to understand the main mechanism of action of α-PFPs. As mentioned in the previous section, this kind of topology is favored by a relative flexibility factor intrinsic to the composition and size of the peptide chain. Likewise, a great body of experimental evidence seems to indicate that toroidal pores are indeed transient, with a half-life of a few milliseconds [114], although some may transition to more stable toroidal pore geometries [115]. Under this optic, it has been proposed that the formation and evolution of those pores is facilitated by the rapid movement of lipids along the pore wall. Consequently, when peptide–lipid ratios are high enough and a certain energetic threshold is exceeded, membranes tend to solubilize as a large number of monomers cooperatively associate to form nanomicelles [116]. However, both the toroidal pore model and the detergent-like micelle formation model are associated with a previous (or even simultaneous) stage in which peptides ‘carpet’ the bilayer surface, although they differ in how the bilayer integrity is altered. In any case, both the formation of toroidal pores and the processes leading to the micellization of the lipid bilayer are a function of the membrane curvature, specifically, the propensity of membranes to acquire positive curvature. Thus, the propensity of lipids to acquire a positive curvature must be coordinated with the propensity of peptides to induce it directly.

It has been shown that, in the case of aurein 2.2 (GLFDIVKKKVVVGALGSL-NH_2_) and aurein 2.3 (GLFDIVKKKVVVGAIGSL-NH_2_) each peptide is able to promote toroidal pore formation by favoring the disruption of acylated chains by inducing a less pronounced positive curvature when the membrane thickness is high (POPC/POPG, 1:1 mol/mol) but inducing nanomicelle formation when the membrane is thinner and, thus, the hydrophobic core is smaller (DMPC/DMPG, 1:1 mol/mol) [117]. These findings suggest that an important parameter to be considered, to establish the fate that an α-PFP might have in its interaction with the lipid bilayer, is its propensity to acquire positive intrinsic curvatures. This propensity, in turn, depends both on the degree of saturation of the acylated chains and on the spontaneous curvature of the lipids as a function of their geometry and the effect that this parameter has in terms of the flexibility that the membrane acquires as a fluid [118,119,120].

**Figure 5:**
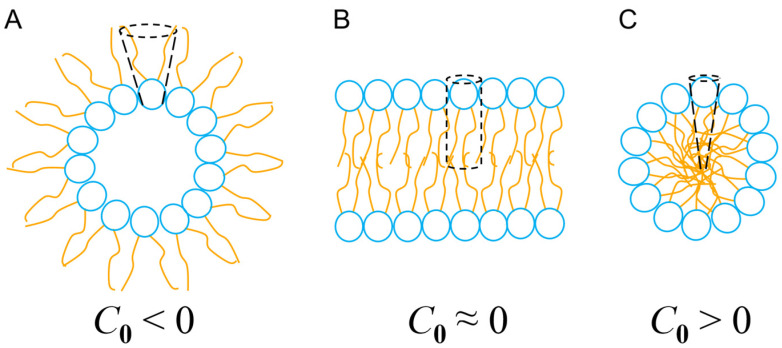
Types of curvature and self-assembly of lipids in nanostructures. (**A**) Inverted micelles, where *C*_0_ < 0 leads to inverted phases. (**B**) Planar lipid bilayer, *C*_0_ ≈ 0. (**C**) Micelles, *C*_0_ > 0. See text for details.

In geometric terms, the shape of a lipid can be calculated by estimating its spontaneous curvature,

*C*_0_ = 2(*V*/*Al* − 1)/*l*,

where *V* denotes the volume of the entire lipid, *l* quantifies its total length and *A* represents the cross-sectional area of the head group (Figure 5). Thus, the geometry of lipids, depending on their chemical composition and their degree of packing, can promote different degrees of flexibility (and fluidity) in the membrane. This membrane fluidity, in the context of the interaction with intrinsically flexible α-PFPs, facilitates (or not) the adaptation of conformers capable of maximizing their configurational entropy and evolving towards the formation of pores with high curvature (and toroidal geometry) or resulting in micellization processes. In the case of aureins 2.2 and 2.3, two peptides that are too short to pass through the bulk of the membrane in a monomeric form, the results of Cheng and cols. [117] could also indicate that thin membranes with a high spontaneous curvature (*C*_0_ > 0) but that are semifluid (rigid) facilitate the formation of micelles. However, thicker membranes with a degree of curvature close to zero (*C*_0_ ≈ 0), and that are, therefore, less prone to generate structures with a high spontaneous curvature (i.e., micelles), besides being more fluid and flexible, could promote the formation of toroidal pores in a cooperative manner.

Unlike barrel-stave pores, toroidal pores exhibit a strongly positively curved geometry and can be considered a state where the lipid bilayer is subject to an excess of packing frustration stabilized by the presence of α-PFPs, however transient. Under such conditions, sterically bulky amino acid residues such as Phe and Trp play a determining role. These residues could act as topological wedges with a certain conformational freedom and that behavior can change their position inside the membrane, thus facilitating the formation of these kind of pores for short periods of time [121]. Consequently, when a certain P/L threshold is exceeded, the tendency to increase the intrinsic curvature parameter, *C*_0_, would be facilitated. This would result in the formation of micelles, creating a solubilizing effect, similar to the action of a detergent.

The effect of spontaneous lipid curvature is critical in the bioactivity of α-PFPs. This is also interesting from an evolutionary perspective, since a large number of peptides are actually as short as about 13-aa, for example, those present in scorpion venoms [107]. Aurein 1.2 (GLFDIIKKIAESF-NH_2_), present in the exudates of frogs of the genus *Litoria*, is one of the most studied and also one of the shortest peptides found in nature. It has been convincingly demonstrated that this peptide is able to form mats on the surface of target model membranes and, thus, promote micellization phenomena [122]. The ‘carpet’ mechanism for these very short peptides could be the most frequent process to permeabilize membranes, since they do not have the theoretical minimum size necessary to cross the lipid bilayer. Even so, the possible formation of more-or-less stable pores through the formation of dimers capable of stabilizing toroidal topologies, in addition to promoting membrane solubilization by micellization, is not ruled out [123,124,125].

## 6. The Mechanical Effect of Flexibility on the Bioactivity of α-PFPs

**Figure 6:**
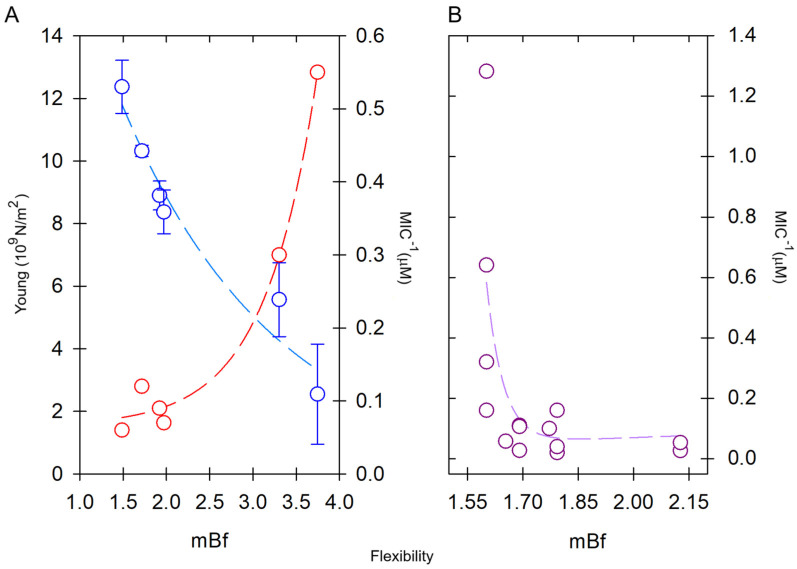
Effect of the intrinsic flexibility on pore-forming peptides with α-helical structure (α-PFPs). (**A**) Young’s modulus (red) is high in rigid peptides but decreases in flexible peptides, which are more active in terms of the MIC (blue). The flexibilities of the peptides are compared in increasing order: Maculatin-1.1 > Brevinin-1 > Pseudin-2 > ranateurin-1 >> HP(2-20) > CAP18. Young’s modulus data were taken from Liu et al. [129]. (**B**) Comparison of the bioactivity of six short scorpion peptides in increasing order of flexibility: VmCT1 > TsAP2 > Stigmurin > Ctriporin > Uy234 > Pin2. In this case, the most active peptides were those with low mean flexibility index (mBf), i.e., rigid. MIC data were reviewed for different bacteria in ref. [107].

The effects of flexibility in terms of vibrations, rotations of specific chemical groups, folding, allosteric regulation and binding to chemical ligands are those typically associated with the function and dynamism in proteins [126]. As mentioned previously, in such short peptides, the flexibility must be even more decisive and define the absolute degree of folding of the peptides. This parameter also reflects the flexural resistance, acting as a mechanical determinant of the antimicrobial actions for peptides that are intrinsically very flexible and probably not very active, or for more rigid peptides, making them instead more active (Figure 6). This principle has been tested on some short peptides present in the venoms of some scorpions, peptides as stigmurin, Ctriporin, VmCT1, TsAP2, Uy234 or Pin2. However, this correlation should always be considered as a function of the bacterial strain studied, since the bioactivity of these peptides often varies between different bacteria, which could suggest the important effect of lipid composition (reviewed in [107]). For example, Pleurocidin (GWGSFFKKAAHVGKHVGKAALTHYL-NH_2_), an intrinsically more rigid peptide (mBf = 1.75) in comparison with magainin 2 (GIGKFLHSAKKFGKAFVGEIMNS-NH_2_) (mBf = 2.03), shows greater conformational freedom in MD simulations, which correlates with its better performance in interacting with lipid bilayers and reflects its antibacterial potency [127].

Aurein 1.2, a very short and rigid peptide (mBf = 2.00) is comparatively less effective compared to an analogous peptide, derived from human LL-37, so-called LLAA (RLFDKIRQVIRKF-NH_2_), whose flexibility is significantly higher (mBf = 2.22) and considerably more active [128]. Likewise, peptides such as Maculatin 1.1, Brevinin-1 and Pseudin-2 are rigid and less active compared to more flexible peptides such as Ranateurin-1, HP(2-20) and CAP18, which are orders of magnitude more active in terms of the MIC [129]. These authors determined, for the first time, the correlation between the degree of stiffness of some short peptides and their Young’s modulus of elasticity and bioactivity against specific bacteria (Figure 6A). It was also determined that the antimicrobial activity associated with rigid peptides is enhanced by slightly increasing their flexibility, while highly flexible peptides increase their activity by making them slightly stiffer [129,130]. Thus, modulation of the intrinsic flexibility of AMPs could prove to be crucial as a structural parameter in their antimicrobial activity (Figure 6B). This type of approach enables the development of attractive mechanistic design methods by generating analogous peptides from sequences found in natural sources, which are either more rigid or more flexible compared to the original sequence.

## 7. C-Terminal Carboxyamidation

The synthesis of short amidated peptides has been described in a large number of metazoans [107,131,132,133,134]. The biological activity of α-PFPs is associated with a characteristic post-transcriptional modification, present mainly in very small peptides (10–30 residues). This modification is a C-terminal amidation, which has been detected in peptides as diverse as melittin, cecropins, PGLa, dermaseptins, PR-39, diptericin, eumenin mastoparan-AF and dermaseptin-S3, among other sequences (reviewed in ref. [135]). In those studies, it has been determined that, in contrast to their carboxylated versions, amidated peptides exhibit greater bactericidal and hemolytic activities (Table 1). It was originally thought that C-amidation promoted an increased attraction to negatively charged membranes, since removal of the C-terminal hydroxyl group increases the net charge of the peptides [131]. However, it has also been established that the contribution of the main-chain positive charge is significantly lower in terms of the transit of the peptides from their surface orientation to a perpendicular position across the lipid bilayer [36].

Instead, a stabilizing role of the α-helical structure has been established as part of the effects of this chemical modification [58,135,136]. The stabilization of α-helical structures must be particularly energetically costly for short sequences (10–25 residues), given the fact that each residue must establish H-bonds every 3.6 positions per turn. Thus, the presence of the amide group, by favoring the formation of additional H-bonds, could contribute to reducing the energetic cost of stabilizing α-helices through networks of H-bonds along the main chain [58].

This stabilizing effect of the amide group is manifested, as in the case of dermaseptin S3 (ALWKNMLKGIGKLAGKAALGAVKKLVGAES), facilitating the formation of peptides up to 30% more ordered, compared to the natural carboxylated forms, which are more flexible and dynamic and less effective against pathogenic microbes under controlled experimental conditions [136,137] (Table 1). An analogous example is that of eumenine mastoparan-AF, whose natural form is the amidated one (INLLKIAKGIIKSL-NH_2_), but when the terminal amide group is removed by its carboxylated counterpart, its bioactivity is also reduced. In addition, the natural peptide (amidated) is more ‘rigid’, i.e., ordered, with a high α-helix content (83%), while the peptide in its carboxylated form exhibits two helical regions (31%) with high conformational freedom and that are dynamically independent of each other. These conclusions were obtained through circular dichroism experiments, NMR and molecular dynamics simulations [135]. The presence of the C-terminal amidation in aurein 2.6 (GLFDIAKKVIGVIGSL-NH_2_) and aurein 3.1 (GLFDIVKKIAGHIAGSI-NH_2_) also facilitates the formation of α-helical structures. This promotes a deeper insertion through the polar heads of the lipids and also facilitates the reorientations of dipoles with the negatively charged groups of lipids such as PC and PS [138]. In addition to this order-promoting role in α-PFPs, C-amidation has been also linked to an increased resistance to degradation by carboxypeptidases and lower turnover rates [139].

**Figure 7:**
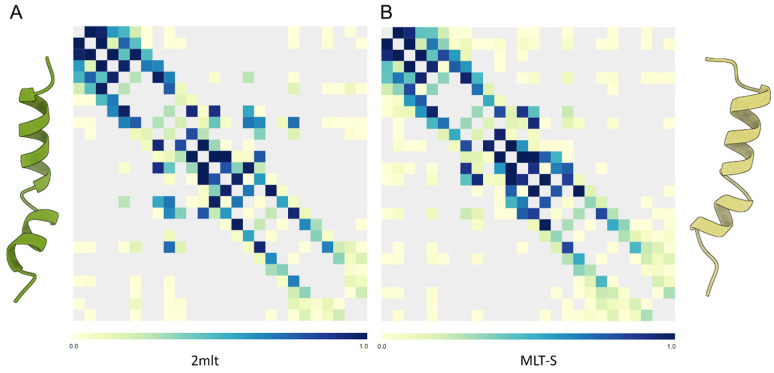
(**A**) Map of intrapeptide contacts for C-amidated melittin (PDB: 2mlt, left) and (**B**) the T10S C-carboxylated variant (MLT-S, right). Contact types are color-coded on the scale shown on each bar. The MLT-S variant shows some middle-distance contacts, which are less frequent in the wild-type peptide, in agreement with its lower intrinsic flexibility.

The optimized bioactivity of amidated AMPs could be also related to an increase in the hydrophobicity of the modified C-terminal end, which would facilitate their partitioning into the hydrophobic core of lipid membranes [140]. The interpretation of these experimental results may not be trivial, since the permeabilizing effect of aurein1.2 (GLFDIIKKIAESF-NH_2_) in synthetic liposomes based on dye-release experiments using DMPC lipids suggests a disruptive effect of this peptide, which has been interpreted in terms of the hydration capacity of the C-terminal end of the wild-type peptide [141]. Thus, the presence of the amide group could be not determinant during the adsorption stage to the lipid bilayer but, once the peptide is bound, this modification could accelerate helix formation. With this advantage, amidated peptides, unlike carboxylated peptides, are better adapted to self-aggregate locally at the membrane interface, facilitating the generation of aqueous pores [142]. Therefore, it seems that the effect of C-amidation on the increase in the potency with which these peptides are able to kill bacteria is of a purely structural nature, as it induces more ordered peptides, reduces their conformational freedom and facilitates their interactions with the phospholipid bilayer. Another interesting aspect linked to these structural effects is found at the compositional level, since in general, amidated peptides are intrinsically more rigid than those that are naturally carboxylated (Figure 7). Thus, the conformational freedom, typical of carboxylated peptides but restricted in amidated peptides, could also be the result of the amino acid composition of these peptides. In this sense, intrinsically rigid peptides are generally more potent against bacteria and show greater hemolytic activity. However, as mentioned above (Section 6) the flexibility parameter is very relative when trying to evaluate its contribution in terms of the bioactivity of this class of peptides. For example, what we know about the potency of Meucin-13 and Meucin-18 must be interpreted with care since Meucin-13 is a relatively more rigid peptide than Meucin-18 but, at the same time, is less effective in terms of its hemolytic activity [143] (Table 1). The fact that both meucines are not equivalent in terms of chemical composition may be part of the answer, since each case should be interpreted in its proper context and the mechanisms of action of each peptide could be very specific in each case, and therefore, not entirely comparable.

In any case, this kind of information is crucial for the optimized design of variants based on the sequences of a plethora of natural peptides. While the study of their interaction with the lipid bilayer is fundamental, the elucidation of their potential efficiency based on the prediction of their conformational freedom and the structural changes that take place in the aqueous milieu, just before the first contact with the membrane surface, is also of utmost importance, as has been suggested elsewhere [32,144,145].

**Table 1:** Primary structures and intrinsic flexibilities of selected α-helical AMPs.

Peptide	Natural Source	Sequence	Flexibility (mBf)	*S. aureus* MIC (mg∙mL^−1^)	Hemolytic Activity (%)	Ref.
**Amidated**						
Meucin-13	*Mesobuthus eupeus*	IFGAIAGLLKNIF-NH_2_	1.58	ND	37.7	[143]
IsCT	*Opisthacanthus madagascariensis*	ILGKIWEGIKSLF-NH_2_	1.97	3	ND	[146]
Mastoparan L	*Vespula lewisii*	INLKALAALAKKIL-NH_2_	1.80	18.5	ND	[147]
Aurein 2.5	*Litoria aurea*	GLFDIVKKVVGAFGSL-NH_2_	1.79	0.05	ND	[148]
Aurein 3.1	*Litoria raniformis*	GLFDIVKKIAGHIAGSI-NH_2_	1.74	0.05	ND	[148]
PGLa	*Xenopus laevis*	GMASKAGAIAGKIAKVALKAL-NH_2_	1.85	16	ND	[76]
Melittin	*Apis mellifera*	GIGAVLKVLTTGLPALISWIKRKRQQ-NH_2_	2.01	ND	95	[149]
Dermaseptin S3	Synthetic	ALWKNMLKGIGKLAGKAALGAVKKLVGAES-NH_2_	2.03	3.02	ND	[137]
Cecropin A	*Anopheles gambiae*	GRLKKLGKKIEGAGKRVFKAAEKALPVVAGVKAL-NH_2_	2.39	0.18–0.35	ND	[150]
Cecropin A	*Hyalophora cecropia*	KWKLFKKIEKVGQNIRDGIIKAGPAVAVVGQATQIAK-NH_2_	2.23	8	ND	[151]
Cecropin D	*Bombyx mori*	GNFFKDLEKMGQRVRDAVISAAPAVDTLAKAKALGQ-NH_2_	2.17	4.6	ND	[152]
Sarcotoxin IA	*Sarcophaga peregrina*	GWLKKIGKKIERVGQHTRDATIQGLGIAQQAANVAATAR-NH_2_	2.11	ND ^a^	ND	[153]
**Carboxylated**						
Hp1470	*Heterometrus petersii*	IFKAIWSGINRLF-COOH	1.70	6.26	ND	[154]
Eumenitin	*Eumenes rubronotatus*	LNLKGIFKKVASLLT-COOH	2.04	9.9–98.7	ND	[155]
Aurein 2.5	*Litoria aurea*	GLFDIVKKVVGAFGSL-COOH	1.79	ND ^b^	ND	[156]
Aurein 3.1	*Litoria raniformis*	GLFDIVKKIAGHIAGSI-COOH	1.74	ND	ND	[148]
Meucin-18	*M. eupeus*	FFGHLFKLATKIIPSLFQ-COOH	1.73	ND	74	[143]
PGLa	Synthetic	GMASKAGAIAGKIAKVALKAL-COOH	1.85	129	ND	[76]
Melittin-S	*A. mellifera*	GIGAVLKVLSTGLPALISWIKRKRQQ-COOH	2.03	ND	35	[149]
Dermaseptin S3	*Phyllomedusa sauvagii*	ALWKNMLKGIGKLAGKAALGAVKKLVGAES-COOH	2.03	30.24	ND	[137]
M-poneritoxin-Nc3a	*Neoponera commutata*	GWKDWLNKAKDFIKEKGPEILRAAANAAIN-COOH	2.33	0.08–0.16	50 ^c^	[157]
U1-poneritoxin-Ng3b	*N. goeldii*	GWKDWLKKGKEWLKAKGPGIVKAALQAATQ-COOH	2.46	ND	ND	[158]
Cecropin A1	*Ae. albopictus*	GGLKKLGKKLEGVGKRVFKASEKALPVAVGIKALGK-COOH	2.55	ND	ND	[159]
Cecropin A	*Aedes aegypti*	GGLKKLGKKLEGAGKRVFNAAEKALPVVAGAKALRK-COOH	2.44	>0.064	2	[160]

^a^ Inhibition data determined only for *E. coli*. ^b^ The -COOH form has lesser antimicrobial potency against *Klebsiella pneumoniae* than the -NH_2_ analogue. ^c^ 0.34 mg/mL.

## 8. Conclusions

The bioactivity of pore-forming peptides, being short sequences, is closely related to their structure, the formation of dipole vectors and their ability to insert into lipid bilayers. However, these peptides are generally disordered prior to their interaction with the membrane. Thus, the role of certain residues capable of conferring specific structural constraints, such as proline, glycine or the modification of the C-terminal end by a specific carboxyamidation, has important repercussions for the stabilization of α-helical structures. Once inserted into the membrane, these peptides evolve their aggregation state towards pores with toroidal, barrel-stave geometries or by carrying out solubilization events through the generation of nanomicelles. Each of these molecular architectures is, in turn, determined by the contribution of the flexibility of the amino acid sequence, the propensity of lipids to spontaneous curvature events and the size of the participating peptides. Establishing a general principle in which the flexibility parameter is the condition for understanding the bioactivity of these peptides is probably an oversimplification of more complex multivariate phenomena. In any case, the study of this class of biomolecules, from a dynamic compositional perspective and in the context of their interaction with biomembranes of a specific composition, is decisive for the optimization of new peptides with particular antimicrobial properties.
